# Nonproteinuric Preeclampsia among Women with Hypertensive Disorders of Pregnancy at a Referral Hospital in Southwestern Uganda

**DOI:** 10.1155/2021/9751775

**Published:** 2021-08-30

**Authors:** Asiphas Owaraganise, Richard Migisha, Wasswa G. M. Ssalongo, Leevan Tibaijuka, Musa Kayondo, Godfrey Twesigomwe, Joseph Ngonzi, Henry Mark Lugobe

**Affiliations:** ^1^Department of Obstetrics and Gynecology, Mbarara University of Science and Technology, Mbarara, Uganda; ^2^Infectious Diseases Research Collaboration, Kampala, Uganda; ^3^Department of Physiology, Mbarara University of Science and Technology, Mbarara, Uganda; ^4^Department of Obstetrics and Gynecology, Mbarara Regional Referral Hospital, Mbarara, Uganda

## Abstract

**Background:**

Preeclampsia is a priority obstetric emergency requiring urgent diagnosis and treatment to avert poor pregnancy outcomes. Nonproteinuric preeclampsia poses even greater diagnostic challenges due to contested diagnostic criteria by the clinical practice guidelines and variable clinical presentation. Previously, preeclampsia was only diagnosed if high blood pressure and proteinuria were present. This study determined the prevalence of nonproteinuric preeclampsia and associated factors among women admitted with hypertensive disorders of pregnancy at a referral hospital in southwestern Uganda.

**Methods:**

Women with hypertensive disorders of pregnancy were consecutively enrolled in a cross-sectional study at Mbarara Regional Referral Hospital between November 2019 and May 2020. We interviewed all pregnant women ≥20 gestation weeks presenting with hypertension and obtained their sociodemographic, medical, and obstetric characteristics. We excluded women with chronic hypertension. We measured bedside dipstick proteinuria in clean-catch urine. Preeclampsia was defined as hypertension plus any feature of severity including <100,000 platelets/ul, creatinine >1.1 g/dl, and liver transaminases ≥twice upper normal limit with or without proteinuria. We defined nonproteinuric preeclampsia in participants with <+2 urine dipstick cut-off and determined the factors associated with nonproteinuric preeclampsia using logistic regression.

**Results:**

We enrolled 134 participants. The mean age was 26.9 (SD ± 7.1) years and 51.5% were primigravid. The prevalence of nonproteinuric preeclampsia was 24.6% (95% CI: 17.9–32.7). Primigravidity (aOR 2.70 95% CI: 1.09–6.72, *p* = 0.032) was the factor independently associated with nonproteinuric preeclampsia.

**Conclusion:**

Nonproteinuric preeclampsia was common, especially among primigravidae. We recommend increased surveillance for nonproteinuric preeclampsia, especially among first-time pregnant women, who may not be detected by the traditional criteria. Obstetrics care providers should emphasize laboratory testing beyond proteinuria, among all women with hypertensive disorders of pregnancy to optimally diagnose and manage nonproteinuric preeclampsia.

## 1. Introduction

Preeclampsia refers to a multisystem disorder characterized by new onset of hypertension and proteinuria or new onset of hypertension and end-organ dysfunction with or without proteinuria in the last half of pregnancy or postpartum [1]. Preeclampsia is a major cause of maternal and perinatal morbidity and mortality in sub-Saharan Africa including Uganda [2–4]. The global prevalence of preeclampsia ranges from 8 to 10% [5], with the majority of the cases (approximately 75%) of preeclampsia presenting with proteinuria [6].

Formerly, preeclampsia diagnosis was based on the presence of hypertension (blood pressure of ≥140/90 mmHg), ≥20 weeks of gestation, and mandatory proteinuria [7, 8], measured commonly as a random urine protein to creatinine ratio ≥0.3 or persistent ≥30 mg/dL or ≥2+ on the dipstick or rarely as a 24-hour urinary excretion >300 mg [9]. However, preeclampsia with adverse maternofetal events may occur before the traditional proteinuria-based criteria are met [10–12]. Early diagnosis and appropriate management of women with preeclampsia reduces and prevents morbidity and mortality associated with the hypertensive disorders in pregnancy [13, 14]. Nevertheless, the heterogeneity in clinical presentation [15] and clinical practice guidelines [16] may delay diagnosis in settings where haematological testing is not readily available, especially when overt proteinuria is not present.

Proteinuria is increasingly being recognized as not a requirement for a diagnosis of preeclampsia [13]. Despite this, proteinuria is still a mandatory diagnostic criterion in the Uganda Clinical Guidelines [7] and other clinical guidelines like for the World Health Organization (WHO) [17], but not for the United States, Canada, and Australia [18]. This discrepancy in the various guidelines about the requirement of proteinuria for a diagnosis of preeclampsia may result in delayed or incorrect diagnosis, yet proper recognition and appropriate classification of preeclampsia contribute to standardized management and improved fetomaternal outcomes [19]. There are limited data on the prevalence of nonproteinuric preeclampsia in sub-Saharan Africa including Uganda, yet these are needed to guide comprehensive screening of pregnant women to improve timely and accurate diagnosis of preeclampsia. Furthermore, the risk profile of women with nonproteinuric preeclampsia, including sociodemographic, medical, and obstetric factors, ought to be understood in order to optimize surveillance, diagnosis, and appropriate management of preeclampsia, for improved outcomes [18]. This study determined the prevalence and associated factors of nonproteinuric preeclampsia among women with hypertensive disorders of pregnancy attending a regional referral hospital in southwestern Uganda.

## 2. Materials and Methods

### 2.1. Study Design, Setting, and Study Population

This was a cross-sectional study among pregnant women with high blood pressure (≥140/90 mmHg) admitted to the antenatal ward of Mbarara Regional Referral Hospital (MRRH) in southwestern Uganda from November 2019 to May 2020. MRRH is a government-funded public tertiary hospital. The hospital conducts approximately 9,000 deliveries per year. The average monthly admission to the antenatal ward is approximately 1,142 women. The hospital's maternal mortality ratio stands high at 375 per 100,000 live births with preeclampsia coming second to hemorrhage [20].

### 2.2. Eligibility Criteria

We included pregnant women ≥20 weeks of gestation with hypertension. Hypertension was defined as blood pressure greater or equal to 140/90 mmHg measured twice at least 4 hours apart, or single reading greater or equal to 160/110 mmHg. We excluded women with chronic hypertension.

### 2.3. Sample Size and Sampling

We used Epi Info (version 7.1.4.0, CDC, Atlanta, US) to compute a sample size of 134 participants using a single population proportion calculation with the following assumptions: 95% confidence level, 5% precision, a design effect of 1, 9% prevalence rate of preeclampsia [5], and a 10% nonresponse rate, from an estimated source population of 3,436 pregnant women. Given the estimated average monthly admission to the antenatal ward of 1,142 women, we estimated the finite accessible study population over the three months proposed by study duration. Consecutive sampling was used to enrol the study participants.

### 2.4. Study Procedures

The trained research assistants (who were midwives) consecutively approached all pregnant women who presented to the antenatal unit of the maternity ward of MRRH, explained the study approach, and invited them to participate. Of women who accepted to be screened, those that fulfilled the study eligibility criteria were enrolled into the study. All pregnant women presenting ≥20 weeks of gestation had a blood pressure measured by research staff on admission. Our blood pressure measurement standard operating procedure was as follows: We used a Visomat® Electronic digital dial blood pressure machine (manufactured by OBL: Visomat, Zum Ottersberg Wertheim am Main, Germany) to measure the blood pressure. A woman was allowed time to rest while seated, without talking for at least 5 minutes. A blood pressure cuff was placed 1-2 cm above the elbow on either arm supported at heart level on a table or a chair armrest, while she was seated leaning back against a chair, without tight clothing around the upper arm and both feet on the floor. Women were instructed not to move, strain, or talk, while the measurement was being taken. The machine was turned on, and the cuff allowed to automatically inflate, deflate, and the display result was taken as the correct woman's blood pressure; the process was repeated upon displaying an “error” reading. We defined hypertension as sustained elevated blood pressure, that is, systolic blood pressure ≥140 mmHg and/or diastolic blood pressure ≥90 mmHg four hours apart, or single reading of severe hypertension is defined as systolic blood pressure ≥160 mmHg and/or diastolic blood pressure ≥110 mmHg. Each woman who screened positive for hypertension underwent further clinical evaluation and laboratory screening; full hemogram, renal function test, and liver enzymes test; and bedside proteinuria measurement to diagnose preeclampsia. The clinical evaluation included history taking, physical examination, and medical records (antenatal card) review.

To measure spot proteinuria, the research assistant gave each woman a labelled sterile urine container and instructed her to collect about 10 ml of clean-catch urine or to collect it at the urine drainage port after removing the urine bag for those who had a urethral catheter in situ. The research assistant immersed Cypress Diagnostics ® colour-coded 10 parameter urine dipsticks (manufactured by Cypress Diagnostics, Hulshout, Belgium) in the sample for 60 seconds and then compared it with container visual colour codes following the manufacturer's instructions on the package insert. The read off result was further classified as nonproteinuric if ≤+1 and proteinuria if ≥+2.

For hematologic tests, ten millilitres of blood sample were drawn from an easily accessible vein on the arm and halved into EDTA and plain vacutainers and taken to the laboratory. The Humastar-200 ® clinical chemistry analyser (HUMAN Biochemica und Diagnostica GmbH, Wiesbaden, Germany) was used to measure the serum creatinine in mg/dL and liver transaminases in IU/L on the 5 ml blood in a plain vacutainer. The other 5 ml in an EDTA vacutainer bottle was run on the Sysmex XN-1000i ® 5-part haematology analyser (Sysmex America, Inc. Lincolnshire, Illinois, USA) to measure haemoglobin concentration in g/dL and platelet count per microliter.

### 2.5. Data Collection and Study Variables

Our primary outcome was nonproteinuric preeclampsia, which was defined in pregnant women ≥20 weeks of gestation with hypertension and at least one severity feature of preeclampsia but without proteinuria. Features of severity were defined as presence of at least any one of the following: (1) unexplained severe headache or blurred vision or coma or convulsion; (2) alanine and aspartate liver transaminases level at least twice of the normal upper limit; (3) thrombocytopenia <100,000/*μ*L; and (4) serum creatinine >1.1 mg/dL.

We interviewed women with hypertension (abstracted some of the data from the woman's clinical record) and captured the data using a pretested-structured questionnaire. The variables recorded included the following: (1) sociodemographic data—age, level of education, marital status, employment status, age, occupation, alcohol use, or smoking; (2) clinical data—chronic hypertension, renal disease, liver disease; unexplained severe headache, visual disturbances, convulsions, epigastric pain, dyspnoea, plus weight, and height measurements; (3) obstetric data—parity, gestational age, multifetal gestation, history of preeclampsia during previous pregnancies, antenatal attendance, and booking blood pressure; and (4) blood test results data—full hemogram, renal function, and liver transaminases.

### 2.6. Ethical Considerations

Ethical approval to conduct this study was obtained from the Mbarara University Research Ethics Committee (Protocol reference number 14/01-19). Written informed consent was obtained from all study participants using consent forms in English and local language-translated versions. All study methods were performed in accordance with the Declaration of Helsinki guidelines and regulations.

### 2.7. Data Management and Analysis

Data were entered into a secure online backed up RECaP® [21] database version 8.2 hosted at Department of Obstetrics and Gynecology at the Mbarara University of Science and Technology (MUST). Statistical analyses were performed using Stata statistical software: Release 14 (StataCorp LP, College Station, Texas, USA).

We computed descriptive statistics and tabulated the baseline participant characteristics as frequency proportions and percentages for categorical variables, or mean with standard deviation for continuous normally distributed variables, compared across the nonproteinuric and proteinuric groups. The prevalence of nonproteinuric preeclampsia was determined as a proportion of participants with nonproteinuria and expressed as a percentage. We used binary logistic regression to determine sociodemographic, obstetric, clinical, and laboratory variables associated with dependent variables. Independent variables with at *p* < 0.2 were entered into a multivariable logistic regression model to determine factors independently associated with nonproteinuric preeclampsia, reporting adjusted odds ratios (aORs) at 95% confidence interval. Factors in the final model with *p* < 0.05 were considered statistically significant.

## 3. Results

During the study period, there were 136 pregnant women admitted with hypertensive disorders of pregnancy. We enrolled 134, and two declined to participate in the study.

Participants' baseline characteristics are shown in Table 1. The mean age of the enrolled participants was 26.9 (SD ± 7.1) years. Women with nonproteinuric preeclampsia were younger, the mean age of 23.7 ± 6.8 years versus 27.1 ± 7.1, *p* *=* 0.019, and primigravidae, 72.7% (*n* = 24) versus 44.55% (*n* = 45), respectively, *p* = 0.005, compared with those with proteinuric preeclampsia.

The prevalence of nonproteinuric preeclampsia was 24.6% (95% CI: 17.9–32.7%, *n* = 33) among the 134 women with hypertensive disorders of pregnancy screened for proteinuria.

### 3.1. Factors Associated with Nonproteinuric Preeclampsia

At multivariable logistic regression analysis, only primigravidity (aOR = 2.70; 95% CI: 1.09–6.72, *p* = 0.032) remained independently associated with nonproteinuric preeclampsia (Table 2).

## 4. Discussion

This study revealed a high prevalence of nonproteinuric preeclampsia among women admitted with hypertensive disorders of pregnancy to the maternity ward of Mbarara Regional Referral Hospital, Southwestern Uganda. Primigravidae were more likely present with nonproteinuric preeclampsia. Our finding compares well to the 24.9% prevalence reported by Pyne et al. [22] from a study conducted in Canada and another by Homer and colleagues conducted at three tertiary hospitals in Australia [6] that reported a 26% prevalence. To the contrary, our reported prevalence is lower than 38% described among patients who presented with an eclamptic fit in the United Kingdom [23]. Given that most of these studies on nonproteinuric preeclampsia were based in high-income countries, our study provides new insights into the burden of nonproteinuric preeclampsia in low-income countries. Our finding thus adds pragmatic evidence to potentially improve maternal and fetal outcomes by detecting preeclampsia cases that would otherwise be missed by the traditional diagnostic criteria [24]. Our data therefore support the notion advanced by the International Society for the Study of Hypertension in Pregnancy (ISSHP) report that proteinuria though highly prevalent in preeclampsia is not mandatory for diagnosis of preeclampsia [11, 25]. Our study has implications on Uganda clinical guidelines, and others yet to adapt to the revised definition of preeclampsia considering that proteinuria may not be present at the time of diagnosis in many cases. Equivocal diagnostic criteria of preeclampsia can significantly, and inherently, impact the timeliness of diagnosis and subsequently care received by women with nonproteinuric preeclampsia in similar settings.

In this study, primigravidae were threefold likely to have nonproteinuric preeclampsia. Being primigravid has been reported to be associated with preeclampsia by Omenya and colleagues at Kisii Teaching and Referral Hospital in Kenya [26]. Broadly, literature reports increased risk of preeclampsia in Europe [27], India [10], and Africa [28] among primigravidae. On the basis of our finding, we recommend increased surveillance for preeclampsia among primigravid women using the revised criteria where proteinuria is not mandatory for diagnosis. This will minimise missed opportunities for detection of preeclampsia and potentially improve maternal and fetal outcomes in our low-resource setting.

While our study informs clinical practice on the prevalence of and factors associated with nonproteinuric preeclampsia at a tertiary hospital in a resource-limited setting, it was not without limitations. First, we measured urine protein once using urine dipstick at admission in keeping with the routine clinical practice in our setting, which might have underestimated the patients with nonproteinuric preeclampsia. Second, the study was conducted at a single site and therefore might not be generalized to other settings outside regional referral hospitals in Uganda and similar sub-Saharan African settings. Finally, at our institution, obstetric ultrasonography is not routinely done to pregnant women; therefore, we may have misclassified fetal growth restriction and placental dysfunction cases of nonproteinuric preeclampsia as only hypertension.

## 5. Conclusion

Our study findings imply that nonproteinuric preeclampsia is common among women with preeclampsia in Uganda, especially among the primigravidae. Indifference in end-organ dysfunction frequency between women with nonproteinuric and proteinuric preeclampsia means that proteinuria alone may not be a pertinent piece of information in the preeclampsia care cascade. We recommend increased surveillance for preeclampsia, especially among first-time pregnant women, who may not be detected by the traditional criteria. There is a need for longitudinal studies to assess the prognostic and clinical implications of nonproteinuric preeclampsia, including maternal and fetal outcomes such as obstetric hemorrhage, mode of delivery, and mortality.

## Figures and Tables

**Table 1:** Demographic and obstetric characteristics of women with hypertensive disorders of pregnancy at Mbarara Regional Referral Hospital, Uganda.

Variable *N* = 134		Total *n* (%)	Nonproteinuric preeclampsia (*n* = 33)	Proteinuric preeclampsia (*n* = 101)	*p* value
Age, years (mean ± SD)		26.9 ± 7.1	23.7 ± 6.8	27.1 ± 7.1	0.019*∗*

Married					0.153
	No	09 (6.7)	04 (12.12)	05 (4.95)	
	Yes	125 (93.3)	29 (87.88)	96 (95.05)	

Occupation					0.476
	Housewife	76 (56.7)	17 (51.52)	59 (58.42)	
	Employed	48 (35.8)	4 (12.12)	06 (5.94)	
	Business	10 (7.5)	12 (36.36)	36 (35.64)	

Education					0.080
	≤Primary	85 (63.4)	15 (45.45)	28 (27.72)	
	Secondary	43 (32.1)	15 (45.45)	70 (69.31)	
	≥Tertiary	06 (4.5)	03 (9.09)	03 (2.97)	

Referred in					0.102
	Yes	85 (63.4)	17 (51.52)	68 (67.33)	
	No	49 (36.57)	16 (48.48)	33 (32.67)	

Parity					0.005*∗*
	Primigravidity	69 (51.5)	24 (72.73)	45 (44.55)	
	Multiparous	65 (48.5)	9 (27.27)	56 (55.45)	

Weeks of gestation					0.274
	<37	70 (52.2)	14 (42.42)	56 (55.45)	
	≥37	64 (47.8)	19 (57.58)	45 (44.55)	

Presenting symptom				0.319	
	Headache	102 (76.1)	23 (69.70)	79 (78.22)	0.319
	Epigastric pain	87 (64.9)	18 (54.55)	69 (68.32)	0.150
	Blurred vision	57 (42.5)	4 (42.42)	43 (42.57)	0.988
	Fit/eclampsia	15 (11.2)	04 (12.12)	11 (10.89)	0.846

Attended antenatal care				0.121	
	<4 times	72 (53.7)	5 (27.78)	74 (27.82)	
	≥4 times	62 (46.3)	13 (72.22)	192 (72.18)	

Preeclampsia history					0.158
	Yes	09 (6.8)	04 (12.12)	05 (5.00)	

Booking blood pressure(mmHg)					0.355
	<130/80	58 (43.28)	12 (36.36)	46 (45.54)	
	≥130/80	76 (56.72)	21 (63.64)	55 (54.46)	

Admission blood pressure(mmHg)					0.109
	<160/110	65 (48.51)	13 (39.39)	52 (51.49)	
	≥160/110	69 (51.49)	20 (60.61)	49 (48.51)	

Serum creatinine mg/dL					0.863
	≤1.09	100 (74.63)	25 (75.76)	75 (74.26)	
	≥1.10	34 (25.37)	8 (24.24)	26 (25.74)	

Transaminases IU/L					0.873
	<80	96 (71.64)	24 (72.73)	72 (71.29)	
	≥80	38 (28.36)	9 (27.27)	29 (28.71)	

Platelet count per uL					0.664
	≥100000	109 (81.34)	26 (78.79)	83 (82.18)	
	<100000	25 (18.66)	07 (21.21)	18 (17.82)	

**Table 2:** Univariable and multivariable analyses for factors associated with nonproteinuric preeclampsia at Mbarara Regional Referral Hospital, Uganda.

Factors *N* = 134	Category	Non-PPE *n* (%)	cOR (95%CI)	*p* value	aOR (95%CI)	*p* value
Age (years)	20–30	12 (36.36)	1		1	
	15–19	14 (42.42)	2.9 (1.11–8.37)	0.040	2.19 (0.81–5.02)	0.059
	>30	7 (21.22)	1.2 (0.41–3.28)	0.770	0.67 (0.22–2.05)	0.479

Gravidity	Multigravida	9 (27.27)	1		1	
	Primigravida	24 (44.55)	3.31 (1.40–7.84)	0.020	2.70 (1.09–6.72)	0.032^*∗*^

Referred in	No	16 (18.18)	1		1	
	Yes	17 (81.82)	1.51 (0.23–3.14)	0.104	0.61 (0.29–1.42)	0.252

Gestation weeks	≥37	19 (69.70)	1		1	
	<37	14 (42.42)	1.69 (0.76–3.73)	0.194	0.60 (0.25–1.44)	0.257

Marital status	Married	29 (87.88)	1.0		1	
	Unmarried	4 (12.12)	2.64 (0.66–10.5)	0.166	1.49 (0.34–6.41)	0.593

PPE: proteinuric preeclampsia, cOR: crude odds ratio, and aOR: adjusted odds ratio.

## Data Availability

Deidentified data sufficient to produce primary study findings will be made available on reasonable request to the Department of Obstetrics and Gynecology, Mbarara University of Science and Technology. Data requests can be submitted to the corresponding author.
